# Cross-Species Epitope Sequence Analysis for Discovery of Existing Antibodies Useful for Phospho-Specific Protein Detection in Model Species

**DOI:** 10.3390/ijms26020558

**Published:** 2025-01-10

**Authors:** Yanhui Hu, Chenxi Gao, William McKenna, Baolong Xia, Majd Ariss, Stephanie E. Mohr, Norbert Perrimon

**Affiliations:** 1 Department of Genetics, Blavatnik Institute, Harvard Medical School, 77 Avenue Louis Pasteur, Boston, MA 02115, USAstephanie_mohr@hms.harvard.edu (S.E.M.); 2 Cell Signaling Technology, 3 Trask Lane, Danvers, MA 01923, USA; majd.ariss@cellsignal.com; 3 Howard Hughes Medical Institute, 77 Avenue Louis Pasteur, Boston, MA 02115, USA

**Keywords:** phospho-specific antibody, *Drosophila*, model organisms, conservation, software tool

## Abstract

Signaling pathways play key roles in many important biological processes, such as cell division, differentiation, and migration. Phosphorylation site-specific antibodies specifically target proteins phosphorylated on a given tyrosine, threonine, or serine residue. The use of phospho-specific antibodies facilitates the analysis of signaling pathway regulation and activity. Given the usefulness of phospho-specific antibodies, a number of collections of these antibodies have been generated, typically for the detection of phosphorylated mammalian proteins. Anecdotal evidence shows that some of these are also useful for the detection of phosphorylated forms of orthologous proteins in model organisms. We propose that anti-phospho-mammalian protein antibody collections comprise an untapped resource for research in other species. To systematically analyze the potential utility of anti-phospho-mammalian protein antibodies in other species, we developed the Cross-species Epitope Sequence Analysis software tool (CESA). CESA identifies and aligns orthologous proteins in model species and then analyzes the conservation of antibody target sites. We used CESA to predict what phospho-specific antibodies in a collection from Cell Signaling Technology (CST) might be useful for studies in *Drosophila melanogaster* and other species. CESA predicts that more than 232 sites on 116 *Drosophila* proteins can potentially be targeted by the antibodies initially developed at CST to detect human, mouse, or rat phosphoproteins.

## 1. Introduction

Post-translational modification is essential for the regulation of many cellular processes. For example, phosphorylation can serve as a molecular switch for signal transduction by making the conformational change adjusting protein–protein binding surfaces or the localization of the target protein [1,2]. The availability of phospho-specific antibodies has revolutionized the study of the signal transduction process by making it possible to track not only the expression level but also the activity of the key players of signaling pathways. Phospho-specific antibodies are generated using peptides containing one or more phosphorylated amino acids, and this process is more difficult than traditional antibody production, which can be achieved by utilizing purified antigens or peptide immunogens. Therefore, compared to the available antibodies, the phosphoantibody resources are more limited. For example, based on the antibodypedia [3], more than 7000 human genes can be targeted by antibodies, while less than 500 human genes can be targeted by phospho-specific antibodies based on the PhosphoPlus database [4,5], the largest phosphoantibody resource. The number dramatically drops for model organisms such as *Drosophila melanogaster*, *Danio rerio*, and *Caenorhabditis elegans*, for which only a handful of phosphoantibodies are available commercially. To facilitate the identification of anti-phosphosite antibodies that might be useful in species other than the original target species, we developed the Cross-species Epitope Sequence Analysis (CESA) software tool to evaluate the conservation of phosphorylated sites between the original species in which it was defined—e.g., human, and another species, such as a model organism species.

## 2. Results

### 2.1. Defining Rules for Predicting Cross-Reactivity of Phospho-Specific Antibodies with Orthologous Proteins in Non-Target Species

We reasoned that for an antibody to be useful in detecting a corresponding phosphosite on an orthologous protein in another species, the amino acid sequence of the phosphosite and the surrounding region must be conserved. However, examples in the literature [6] and as reported in commercial antibody catalogs (e.g., selected examples in Table 1) suggest that the region of conservation surrounding the phosphosite can be as small as six amino acids.

To more precisely infer what amino acid identity and similarity is sufficient to predict cross-reaction of an anti-phosphosite antibody directed against ‘protein A’ with some orthologous ‘protein B’ in another species, we first analyzed attributes of anti-phosphosite antibodies already reported to cross-react with another species. As a source for information, we turned to the PhosphoSitePlus web-based bioinformatics resource (https://www.phosphosite.org) [4,5], which is made available by Cell Signaling Technologies (CST), one of the commercial sources for anti-phosphosite antibodies. The March 2024 PhosphoSitePlus release file lists 378,971 phosphosites annotated in 27 organisms, 2357 of which are reported to be recognized by CST antibodies. Among the antibodies targeting these 2357 sites, 12 antibodies are reported in PhosphoSite Plus to cross-react with phosphosites on eight *Drosophila* proteins (Table 1). There are a few other commercial sources with a limited number of anti-phosphosite antibodies that can cross-react with *Drosophila* as well. However, the information regarding the target site is quite limited; for example, the protein accession and the peptide sequence information are not publicly available. Therefore, we only focused on the antibodies from CST. After we aligned the original target sites with seven amino acids on each side with the protein of *Drosophila* ortholog, we evaluated the conservation for these 12 validated sites of cross-reactivity. As expected, the aligned sites are conserved between human and *Drosophila* orthologs, with identical sequences over 6–15 amino acid regions spanning the phosphosite region.

### 2.2. Development of the Cross-Species Epitope Sequence Analysis (CESA) Software Tool

**Figure 1:**
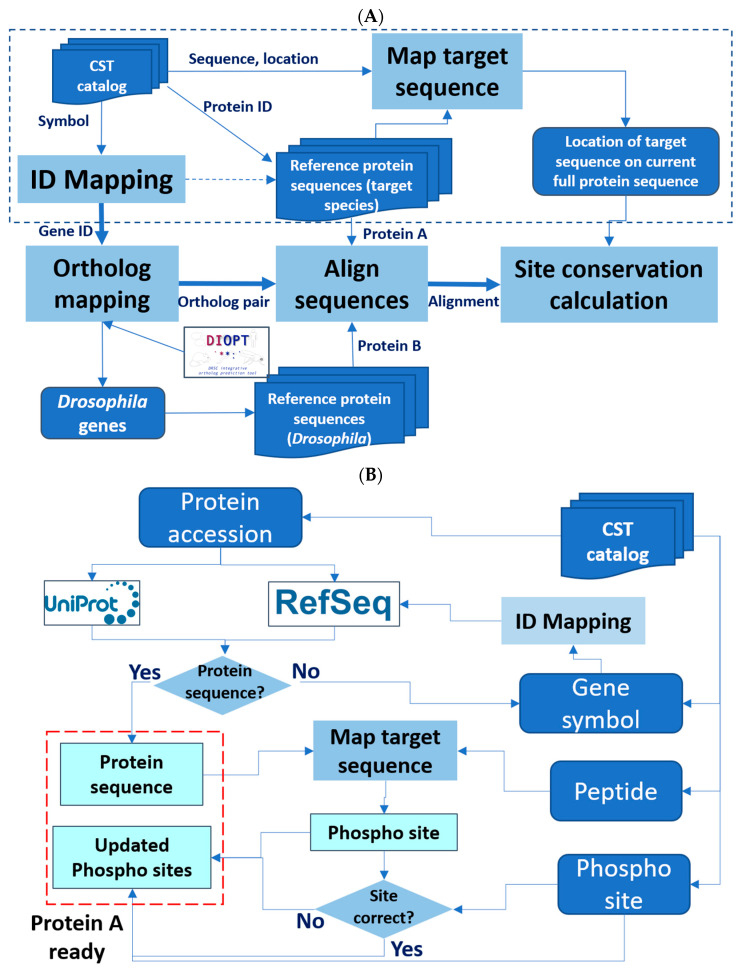
Design of the Cross-species Epitope Sequence Analysis (CESA) software tool. (**A**) Overall workflow. Protein A, original target. Protein B, putative orthologous target identified in another species. An abbreviated presentation of steps needed to ready ‘protein A’ for analysis is shown within the dotted line box. Dark blue represents input and output lists, while light blue represents major steps. (**B**) A detailed presentation of the processing steps needed to ready ‘protein A’ for analysis.

We developed the Cross-species Epitope Sequence Analysis (CESA) software tool to evaluate the conservation of phosphorylated sites between the original species in which it was defined, e.g., human, and another species, such as a model organism species, with the ultimate goal of identifying anti-phosphosite antibodies that might be useful in species other than the original target species. There are five major steps in the pipeline (Figure 1A). First, CESA maps the epitope sequence to a current protein release, such that the full protein sequence can be identified for alignment and the amino acid position of the target site relative to the reference protein sequence is noted. This step of validating the information about the target site on the source protein against which the antibody was originally generated is critical for the downstream analysis. However, we observed that some of the protein accession numbers in the CST catalog are outdated and, therefore, failed to retrieve the full protein sequence from current protein resources. To overcome this issue, we added an additional process for records that failed the first step to identify the current protein records from the RefSeq database using the gene symbol provided in the catalog as well as the gene2refseq mapping file from NCBI (https://ftp.ncbi.nlm.nih.gov/gene/DATA/). Then, these protein sequences retrieved based on gene symbol are compared with the phosphopeptide sequence from the catalog to annotate the location of the phosphosite (Figure 1B). Second, CESA maps gene symbols and/or protein accession numbers to NCBI Entrez Gene identifiers using the gene identifier mapping tools developed in-house. This allowed us to assign Entrez Gene IDs, which are needed to query orthologous genes using DIOPT, an ortholog mapping database that integrates ortholog mapping from more than twenty algorithms [7]. Third, CESA maps input genes from humans or mice to orthologs in the second species, such as *Drosophila*, based on ortholog predictions from DIOPT, with the filter selecting only the mapping of high or moderate confidence [7]. Next, CESA performs pairwise protein alignment of each input protein sequence with all isoforms of the model species ortholog using a global alignment algorithm. In the last step, a parser was developed to identify the target sequence of the input protein and the aligned *Drosophila* sequence, then analyze the conservation of all peptides six to eleven amino acids long spanning each amino acid that can be phosphorylated to identify the sites that are identical between the two species. We allow only one conserved amino acid replacement at the location not right next to the target amino acid (S/T/Y). The amino acid conservation in the area immediately surrounding and including the aligned phosphorylation site with different peptide length cutoff is summarized in the output file.

### 2.3. Application of CESA to Predict Drosophila Targets of Phosphosite Antibodies

**Figure 2:**
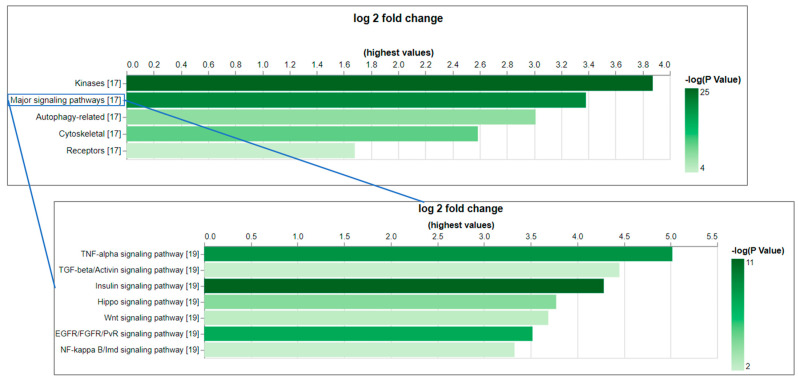
The gene set enrichment analysis (GSEA) of the *Drosophila* genes that can be potentially targeted by CST phospho-antibodies indicates the over-representation of core components of major signaling pathways. GSEA was conducted using PANGEA. The gene sets annotated by GLAD and PathOn (signaling pathway core components) were selected. The enrichment result was filtered using a *p*-value of 0.05 as a cutoff. On the bar graphs from PANGEA, the height reflects fold enrichment while the darkness reflects the negative log10 *p*-value of enrichment.

As a test case, we applied the CESA pipeline to identify anti-phosphosite antibodies at CST that might cross-react with an orthologous protein in *Drosophila*. As an input set, we selected the subset of 2301 phosphorylated sites in PhosphoSitePlus that were originally annotated for humans, mice, or rats and can be targeted by antibodies in the CST collection. Using CESA, 998 of 1170 genes from humans, mice, and rats were mapped to 659 *Drosophila* genes, among which 232 phosphorylation sites on 116 *Drosophila* genes that can be potentially targeted by the CST antibodies originally developed for other species using six amino acids as cut-off (Table 2, Table 3 and Table S1). Among the 116 *Drosophila* genes that can be potentially targeted by CST antibodies, there are only three unannotated genes with CG number as the gene symbol, and 87 (75%) of them have more than 20 associated publications (Table S1). Gene set enrichment analysis was conducted using PANGEA (PAthway, Network and Gene-set Enrichment Analysis) [8] to identify the gene groups that are over-represented among them, and the top gene groups are kinases (47 genes, P adj = 2.80 × 10^−24^) and core components of major signaling pathways (29 genes, P adj = 1.21 × 10^−20^) (Figure 2, Table S2). For example, the *Drosophila* insulin receptor (InR, FBgn0283499) is one of the core components of the insulin signaling pathway. The region spanning the three C-terminal tyrosines (Y1545/1549/1550 on the RB isoform) of this gene not only shares 100% identity with humans but also the phosphorylation at these sites has been observed in *Drosophila* based on the phospho-proteomics data in iProteinDB [9] (Figure S1). The Abcam antibody ab62321 has been tested in humans for the first site, while the CST antibody CST3024 has been tested in humans, mice, and rats for the second and third sites. Based on the literature, Abcam antibody ab62321 has been tested, and its reactivity in *Drosophila* S2R+ cells was shown upon insulin treatment, which activates the insulin signaling pathway [6]. Therefore, it is very likely the CST antibody CST3024 can also target the phosphorylated *Drosophila* protein. Indeed, using the same protocol for S2R+ cell handling, we tested antibody CST3024, and the result showed reactivity as well (Figure S1). It is reasonable to suspect that the antibodies identified by CESA will be important reagents for *Drosophila* research (Table S1).

In addition, we ran CESA for zebrafish (*D. rerio)*, frogs (*X. tropicalis*), mosquitoes (*A. gambiae)*, and worms (*C. elegans*) and identified between 584 and 75 genes that can be potentially be targeted by the current CST collection (Table 2 and Table 3). We observed that a larger portion of phospho-antibodies work across species between genetically closer species, such as 57% of human phosphosites conserved with zebrafish, compared to only 17% of human phosphosites conserved with *Drosophila* (Table 2 and Table 3). Nevertheless, CESA systematically identifies the potential reagents from the existing collection, which is significantly more than the reagents currently available commercially. Altogether, our analysis should advance research in relevant communities, particularly for species with very limited antibody resources.

## 3. Methods

### 3.1. Tool Development

The tool was developed in Python, and DIOPT (*Drosophila* RNAi Screening Center Integrative Ortholog Prediction Tool) release 9 was used to map human, mouse, and rat genes to *Drosophila* genes using a filter to select only orthologous relationships with high and moderate ranks [7]. The tool communicates to the database using the SQLAlchemy package. The pandas package was used to process the query results. Protein global alignment was conducted using the pairwise2 package. The results were then filtered by selecting the longest isoform of each *Drosophila* ortholog gene (Table S1). We also implemented a standalone version of the program that enables local analysis without the need to connect to any database. This program allows the user to compute the result based on the choice of the model organism as well as the choice of window size of the phosphosite specified by the user. This program is publicly available and can be run using a customized catalog file.

### 3.2. Information Retrieval

The March 2024 release of the CST catalog file (Phosphorylation_site_dataset.gz) was obtained from https://www.phosphosite.org/staticDownloads.action; accessed on 22 April 2024). Ortholog mapping information along with NCBI Entrez Gene and RefSeq protein sequences were obtained from DIOPT release 9; accessed on 20 June 2024). Uniprot2geneid mapping was obtained from the UniProt knowledgebase (https://ftp.uniprot.org/pub/databases/uniprot/knowledgebase/idmapping/by_organism/; accessed on 22 April 2024). Human, mouse, and rat protein sequences for UniProt records were obtained from UniProtKB (https://www.uniprot.org/uniprotkb; accessed on 22 April 2024).

### 3.3. Antibody Testing

5 × 10^6^ S2R+ cells per well were plated into a 6-well plate in Schneider’s media supplemented with 10% FBS (fetal bovine serum) overnight. Media was aspirated and replaced with Schneider’s media containing 5 µg/mL insulin for 10 min. Cell pellets were lysed using a lysis buffer containing protease and phosphatase inhibitors on ice for 10 min. Protein concentrations were measured by a BCA protein assay (Thermo Fisher Scientific, Waltham, MA, USA). 2-mercaptoethanol (final, 5%) and 4× Laemmli Sample Buffer were added to protein solutions, and the mixtures were boiled for 10 min. The protein sample was loaded into each lane, and the separation of proteins was performed in a 4–20% gradient Mini-PROTEAN TGX Stain-Free Precast Gel (Bio-Rad, Hercules, CA, USA) using Novex™ Tris-Glycine SDS Running Buffer (Thermo Fisher Scientific). Proteins were transferred from the gel to Immobilon^®^ -FL PVDF Membrane (Millipore) with Pierce™ 10X Western Blot Transfer Buffer (Thermo Fisher Scientific) using a Trans-Blot^®^ Turbo™ Transfer System (Bio-Rad) at 0.4 A, 25 V for 16 min. The membrane was blocked with 5% BSA in TBST for 1 h at room temperature. The membrane was subsequently incubated overnight at 4 °C with Phospho-IGF-I Receptor β (Tyr1135/1136)/Insulin Receptor β (Tyr1150/1151) (19H7) Rabbit mAb #3024 (Cell Signaling Technologies, Danvers, MA, USA) diluted 1:1000 in 5% BSA in TBST. The membrane was washed three times for 3 min with TBST before incubation with Goat anti-Rabbit IgG (H+L) Highly Cross Adsorbed Secondary Antibody, Alexa Fluor™ Plus 800 (Thermo Fisher Scientific) at a 1:2000 dilution and anti-Actin-Rhodamine at a dilution of 1:2500. The western blot was visualized using ChemiDoc MP Imaging System (Bio-Rad).

### 3.4. Code Availability

The standalone version of the CESA program is available on GitHub (https://github.com/chenxi-gao/antibody_discovery; accessed on 19 August 2024).

## 4. Discussion

Antibodies targeting the phosphorylated peptide of endogenous protein are crucial to studying the signaling pathways for processes like cell division and migration, but developing such reagents is both labor- and time-consuming—such resources are quite limited for the majority of model organisms. While current commercial collections were originally designed for mammalian proteins, they may also be useful for detecting phosphorylated proteins in other organisms. To explore this potential, we developed the CESA tool to help researchers identify such reagents. Although we mainly used *Drosophila* as an example in this study, the CESA approach can be applied to any other model and non-model organisms. It is worth noting that since CESA considers all isoforms in the second species, it can predict multiple bands on western blots if conserved sites are identified in more than one protein isoform of different lengths. It is also possible to configure CESA to predict cross-gene activity based on paralog relationships. In addition, CESA can be adapted for other applications, such as analyzing the epitope sequences of regular (non-phospho-specific) antibodies. In summary, we systematically evaluated current CST phospho-antibody to predict the cross-species activity in *Drosophila* and a few other organisms and identified a significant number of new potential useful reagents for species with very limited antibody resources. The result will likely advance research in relevant communities.

## Figures and Tables

**Table 1:** Commercial antibodies against mammalian proteins with validated reactivity in *Drosophila*.

Human Gene	Fly Gene	Human Site +/−7 AA	Fly Site +/−7 AA	Conserved Region (Continues AA)
AKT1	Akt1	RPHFPQFSYSASGTA	EPL FPQFSY QGDMAS	6
PRKAA1	AMPKalpha	SDGEFLRTSCGSPNY	L DGEFLRTSCGSPNY	14
MAPK10	bsk	AGTSFMMTPYVVTRY	AGT T FMMTPYVVTRY	11
MAPK10	bsk	TSFMMTPYVVTRYYR	T T FMMTPYVVTRYYR	13
EIF2S1	eIF2alpha	MILLSELSRRRIRSI	MILLSELSRRRIRSI	15
MAPK12	p38a	RQADSEMTGYVVTRW	R PTEN EMTGYV A TRW	6
MAPK12	p38a	ADSEMTGYVVTRWYR	TEN EMTGYV A TRWYR	6
MAPK1	rl	HDHTGFLTEYVATRW	HDHTGFLTEYVATRW	15
MAPK1	rl	HTGFLTEYVATRWYR	HTGFLTEYVATRWYR	15
RPS6KB1	S6k	NQVFLGFTYVAPSVL	N LI F Q GFTYVAPS I L	8
EIF4EBP1	Thor	PPGDYSTTPGGTLFS	M P EV YS S T PGGTL Y S	6
EIF4EBP1	Thor	GGTLFSTTPGGTRII	GGTL Y STTPGGT KL I	7

Note: red font indicates the target site. Yellow shade highlights different amino acids, while blue shade highlights the same amino acids.

**Table 2:** Summary of phosphosites reported in PhosphoSitePlus, availability of corresponding CST antibodies, and their conservation with *Drosophila*, zebrafish, frogs, mosquitoes, and *C. elegans*.

Species	Sites	Genes	Sites with Antibody	Genes with Antibody	Sites Conserved in Fly	Sites Conserved in Frog	Sites Conserved in Fish	Sites Conserved in Mosquito	Sites Conserved in Worm
human	240,118	17,467	1019	485	187	658	599	181	114
mouse	105,223	13,475	774	407	171	514	485	160	107
rat	31,721	7261	508	278	116	328	311	111	72
other	1893	676	56	33	NA	NA	NA	NA	NA

**Table 3:** Known or predicted phosphosites in *Drosophila,* zebrafish, frogs, mosquitoes, and *C. elegans* that can potentially be targeted using CST antibodies developed to target phosphosites in mammals.

Cut-Off of Conserved Region (aa)	Fly *D. melanogaster*	Frog *X. tropicalis*	Zebrafish *D. rerio*	Mosquito *A. gambiae*	Worm *C. elegans*
6	115	472	599	97	76
7	111	458	578	91	73
8	105	446	564	87	70
9	97	431	544	83	68
10	92	418	518	77	67
11	88	399	495	73	67

## Data Availability

The standalone version of the CESA program is available on GitHub (https://github.com/chenxi-gao/antibody_discovery) (19 August 2024).

## Supplementary Materials

The following supporting information can be downloaded at: https://www.mdpi.com/article/10.3390/ijms26020558/s1.
