# Supplementary material for: Cross-Species Epitope Sequence Analysis for Discovery of Existing Antibodies Useful for Phospho-Specific Protein Detection in Model Species
Source: Int J Mol Sci. 2025 Jan 10;26(2):558. doi: 10.3390/ijms26020558 (PMC11765086; doi:10.3390/ijms26020558)

Supplementary Figure 1. Example CESA prediction of a phospho-antibody likely to cross-react with an orthologous target site in *Drosophila*. Multiple sequence alignment was obtained from iProteinDB with PTM sites (based on phospho-proteomics data) marked using red font. Bottom, alignment of a 60-amino acid region of the human gene IGF1R (1135-1194 on NP_001138093.1) and its orthologs in *mouse musculus* (mm), *rattus norvergicus* (rn), *Xenopus tropicalis* (xt), *Zebra Danio* (dr), *Drosophila melanogater* (dm), and *C. elegans* (ce) that surrounds three tyrosine phosphosite known to be present in the human protein (red box and arrows). Symbols below the alignment indicate amino acids that are identical (*) or similar (: or .) in all of these species to the corresponding amino acid in the human protein. The Abcam antibody ab62321 is reported to recognize phospho Y1135 (1st red box) while CST antibody #3024 is reported to recognize the other two sites (2nd red box) on human protein and we tested CST#3024 . Up left is the screenshot from Abcam for ab62321 while up right is the screenshot from CST about the antibody CST3024. Abcam antibody ab62321 has been tested and shown reactivity in *Drosophila* S2R+ cells upon insulin treatment, which activates Insulin signaling pathway (middle) (Xia et al, PMID: 37078570). We tested and confirmed the reactivity of CST3024 in *Drosophila* S2R+ cells upon insulin treatment as well (up middle).

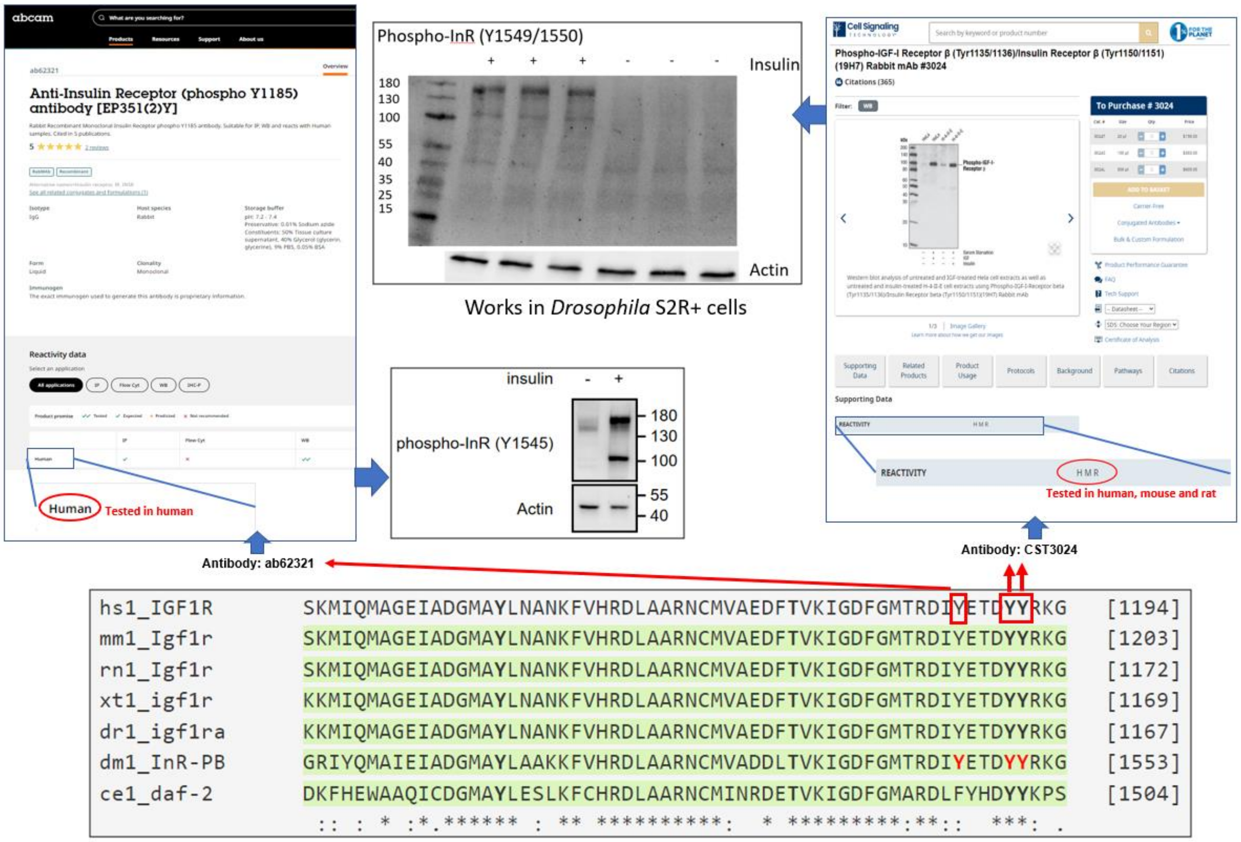

Supplement: Supplementary file 1 [file ijms-26-00558-s001.zip › Figure S1.pdf]
